# Supplementary material for: 27-Hydroxymangiferolic Acid Extends Lifespan and Improves Neurodegeneration in Caenorhabditis elegans by Activating Nuclear Receptors
Source: Molecules. 2025 Feb 21;30(5):1010. doi: 10.3390/molecules30051010 (PMC11902184; doi:10.3390/molecules30051010)
Supplement: Supplementary file 1 [file molecules-30-01010-s001.zip › molecules-3445287-supplementary.pdf]

Supplementary Materials for

27-Hydroxymangiferolic Acid Extends Lifespan and Improves Neurodegeneration in *Caenorhabditis elegans* by Activating Nuclear Receptors

Xiaoyan Gao [1,†], Jing Yu [1,†], Yin Li [2,†], Hang Shi [1], Lijun Zhang [1], Minglv Fang [1], Ying Liu [1], Cheng Huang [1,*] and Shengjie Fan [1,*]

[1] School of Pharmacy, Shanghai University of Traditional Chinese Medicine, Shanghai 201203, China; gxy170402310@outlook.com (X.G.); 401325yj@shutcm.edu.cn (J.Y.); kyshihang@126.com (H.S.); lijunzhang96@163.com (L.Z.); fangminglv@163.com (M.F.); lydiaaaaa_liu@163.com (Y.L.)

[2] School of Agriculture and Medicine, Hebei Open University, Shijiazhuang 050080, China; liyin@hebnetu.edu.cn

* Correspondence: chuang@shutcm.edu.cn (C.H.); shengjiefan@shutcm.edu.cn (S.F.); Tel.: +86-21-51323194 (S.F.); Fax: 86-21-51322194 (S.F.)

† These authors contributed equally to this work.

This Supplementary Materials includes:

Supplementary Figures S1 to S4

Supplementary Tables S1 to S8

Supplementary Figure S1

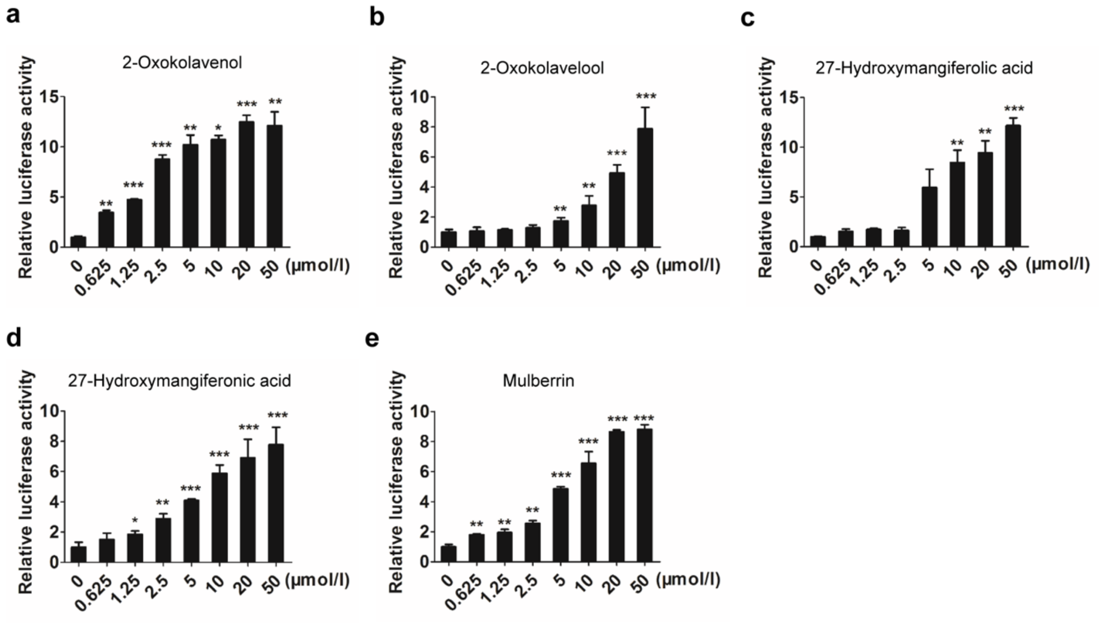

Figure S1. Compounds increase the transcriptional activity of FXR. (a) 2-Oxokolavenol activated FXR transactivity. (b) 2-Oxokolavelool activated FXR transactivity. (c) 27-Hydroxymangiferolic acid activated FXR transactivity. (d) 27-Hydroxymangiferonic acid activated FXR transactivity. (e) Mulberrin activated FXR transactivity. All transcriptional activities of FXR were determined using dual luciferase reporter assays. All data were presented as mean ± S.E.M. Compared with vehicle group, * $p < 0.05$, ** $p < 0.01$, *** $p < 0.001$.

Supplementary Figure S2

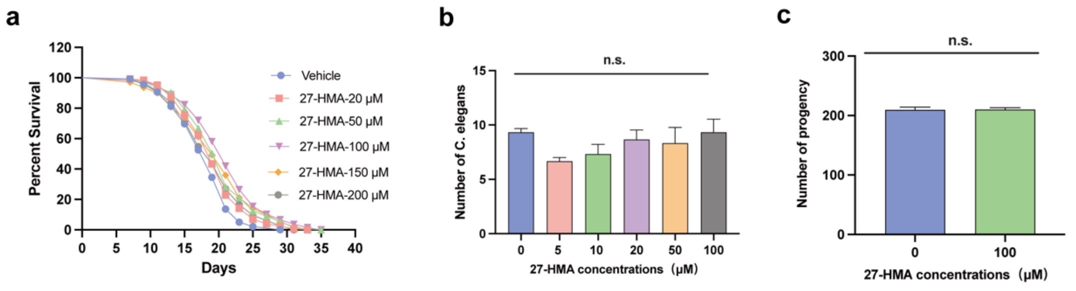

Figure S2. 27-HMA extends lifespan in *C. elegans*. (a) Survival curves of wild type nematodes treated 20, 50, 100, 150 and 200 μM 27-HMA. (b) Chemotaxis of nematodes to E. coli OP50 of each group used as a food resource. (c) The number of total progeny of 27-HMA-treated and untreated nematodes. All data were presented as mean ± S.E.M. Compared with vehicle group, n.s. means no significant difference.

Supplementary Figure S3

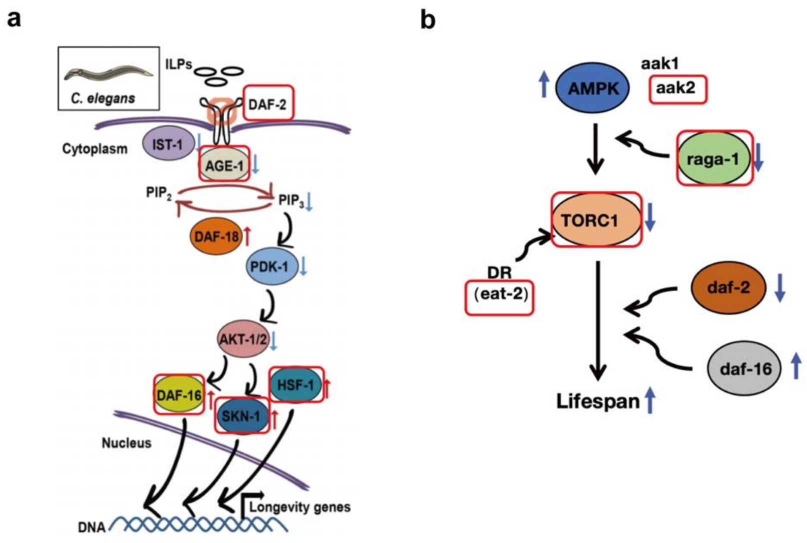

Figure S3. The schematics of signaling pathways. (a) The schematic of IIS signaling pathway. (b) The schematic of TORC1 signaling pathway.

Supplementary Figure S4

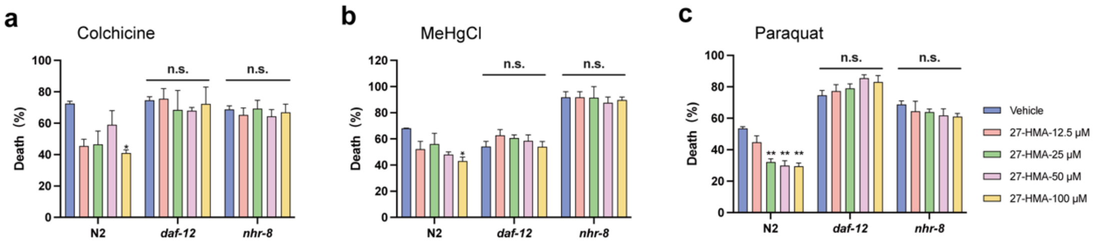

Figure S4. Xenobiotic detoxification may be required for 27-HMA induced longevity. (a) The livability of wild-type, *daf-12 (rh61rh411)* and *nhr-8 (tm1800)* exposed to 4 mM colchicine. (b) The livability of wild-type, *daf-12 (rh61rh411)* and *nhr-8 (tm1800)* exposed to 2 μM MeHgCl. (c) The livability of wild-type, *daf-12 (rh61rh411)* and *nhr-8 (tm1800)* exposed to 200 mM paraquat. All data were presented as mean ± S.E.M. Compared with vehicle group, n.s. means no significant difference, ** $p < 0.01$.

Supplementary Table S1. List of FXR agonists screened.

Compounds	Molecular Formula	Structure
2-Oxokolavenol	$C_{20}H_{32}O_2$	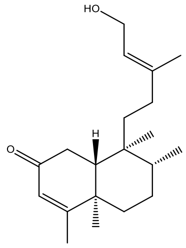

2-Oxokolavelool	$C_{20}H_{32}O_2$	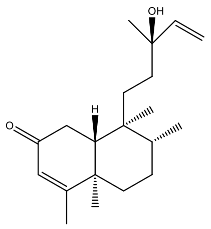
27-Hydroxymangiferolic acid	$C_{30}H_{48}O_4$	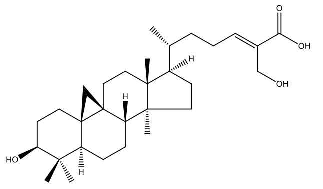
27-Hydroxymangiferonic acid	$C_{30}H_{46}O_4$	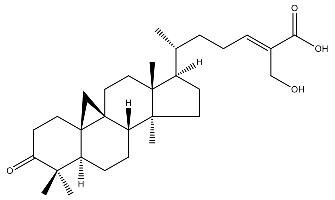
Mulberrin	$C_{25}H_{26}O_6$	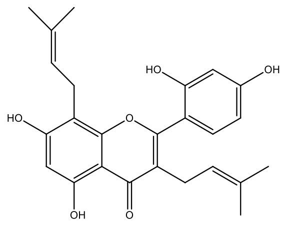

Supplementary Table S2. Summary of the effects of 27-HMA on the lifespans of N2 *C. elegans*.

Strains	Group	Number	Mean Survival ± SEM (days)	Maximum longevity (days)	Median survival time (days)	Increase in mean survival (%)	Increase in maximum longevity (%)	p value of mean survival
N2	Vehicle	139	17.935±0.361	29	19	/	/	/
	27-HMA -20 μM	127	19.252±0.411	33	19	7.34	13.79	P < 0.05*
	27-HMA -50 μM	150	20.227±0.427	35	21	12.78	20.69	P < 0.001***
	27-HMA -100 μM	131	20.924±0.489	35	21	16.67	20.69	P < 0.001***
	27-HMA -150 μM	142	19.622±0.504	33	19	9.41	13.79	P < 0.001***
	27-HMA -200 μM	115	19.017±0.489	31	19	6.03	6.90	P < 0.05*

Notes: Lifespan experiments were analyzed using Kaplan-Meier survival analysis and compared among groups, scoring for significance using the log-rank test. All data were expressed as mean± SEM. *p < 0.05, ***p < 0.001 vs. control group.

Supplementary Table S3. Effects of 27-HMA on the lifespans of N2, *nhr-8*, *daf-12* mutant *C. elegans*.

Strains	Group	Number	Mean Survival ± SEM	Maximum longevity	Median survival time	Increase in mean	Increase in maximum	p value of mean survival

			(days)	(days)	(days)	survival (%)	longevity (%)	
N2	Vehicle	139	17.273±0.472	30	17	/	/	/
	27-HMA-100 μM	121	20.331±0.577	38	21	17.70	26.67	P < 0.001***
nhr-8	Vehicle	112	18.196±0.531	34	19	/	/	/
	27-HMA-100 μM	122	19.180±0.496	30	19	5.40	-11.76	P > 0.05
daf-12	Vehicle	103	15.767±0.514	30	15	/	/	/
	27-HMA-100 μM	112	15.929±0.439	32	17	1.03	6.67	P > 0.05

Notes: Lifespan experiments were analyzed using Kaplan-Meier survival analysis and compared among groups, scoring for significance using the log-rank test. All data were expressed as mean± SEM. ***p < 0.001 vs. control group.

Supplementary Table S4. Effects of 27-HMA on the lifespans of N2, *daf-16*, *daf-2*, *age-1*, *skn-1*, *hsf-1* mutant *C. elegans*.

Strains	Group	Number	Mean Survival ± SEM (days)	Maximum longevity (days)	Median survival time (days)	Increase in mean survival (%)	Increase in maximum longevity (%)	p value of mean survival
N2	Vehicle	136	17.824±0.434	30	17	/	/	/
	27-HMA-50 μM	177	20.458±0.339	36	21	18.48	20.00	P < 0.001***
daf-16	Vehicle	109	16.00±0.341	23	16	/	/	/
	27-HMA-50 μM	104	15.202±0.395	24	16	-0.33	4.36	P > 0.05
daf-2	Vehicle	144	37.764±0.889	55	39	/	/	/
	27-HMA-100 μM	188	38.032±0.741	52	39	0.71	-5.45	P > 0.05
age-1	Vehicle	103	28.592±0.657	43	29	/	/	/
	27-HMA-100 μM	107	29.617±0.709	45	29	3.58	4.65	P > 0.05
skn-1	Vehicle	156	16.333±0.447	25	17	/	/	/
	27-HMA-100 μM	143	16.993±0.450	28	19	4.04	12.0	P > 0.05
hsf-1	Vehicle	146	12.000±0.351	26	11	/	/	/
	27-HMA-100 μM	213	12.160±0.249	26	11	1.33	0	P > 0.05

Notes: Lifespan experiments were analyzed using Kaplan-Meier survival analysis and compared among groups, scoring for significance using the log-rank test. All data were expressed as mean± SEM. ***p < 0.001 vs. control group.

Supplementary Table S5. Effects of 27-HMA on the lifespan of N2 *C. elegans* under conditions of 35°C and H_2O_2.

Strains	Group	Number	Mean Survival ± SEM (hours)	Maximum longevity (hours)	Median survival time (hours)	Increase in mean survival (%)	Increase in maximum longevity (%)	p value of mean survival
N2 (35°C)	Vehicle	59	6.373±0.284	12	6	/	/	/
	27-HMA-100 μM	53	6.868±0.307	12	6	7.77	0	P > 0.05
N2	Vehicle	63	2.794±0.96	5	3	/	/	/

(H_2O_2)	27-HMA-100 μM	48	3.146±0.126	5	3	12.60	0	P < 0.05*

Notes: Lifespan experiments were analyzed using Kaplan-Meier survival analysis and compared among groups, scoring for significance using the log-rank test. All data were expressed as mean± SEM. *p < 0.05 vs. control group.

Supplementary Table S6. Effects of 27-HMA on the lifespans of N2, *let-363*, *raga-1*, *aak-2*, *eat-2*, *sir-2.1*, *fat-2*, *fat-3* mutant *C. elegans*.

Strains	Group	Number	Mean Survival ± SEM (days)	Maximum longevity (days)	Median survival time (days)	Increase in mean survival (%)	Increase in maximum longevity (%)	p value of mean survival
N2	Vehicle	108	16.389±0.371	29	17	/	/	/
	27-HMA-100 μM	143	19.573±0.457	37	19	19.42	27.59	P < 0.001***
let-363	Vehicle	144	18.347±0.509	32	20	/	/	/
	27-HMA-100 μM	143	19.196±0.514	30	18	-0.82	-6.25	P > 0.05
raga-1	Vehicle	125	25.120±0.750	47	25	/	/	/
	27-HMA-100 μM	138	24.377±0.642	47	23	-2.96	0	P > 0.05
aak-2	Vehicle	134	15.306±0.286	21	16	/	/	/
	27-HMA-100 μM	102	14.471±0.351	21	14	-5.46	0	P > 0.05
eat-2	Vehicle	133	27.068±0.703	51	28	/	/	/
	27-HMA-100 μM	133	26.218±0.755	52	26	-3.14	1.96	P > 0.05
sir-2.1	Vehicle	134	19.530±0.392	27	21	/	/	/
	27-HMA-100 μM	141	19.709±0.396	27	21	0.92	0	P > 0.05
fat-2	Vehicle	202	17.772±0.295	27	19	/	/	/
	27-HMA-100 μM	134	17.239±0.394	27	17	-2.99	0	P > 0.05
fat-3	Vehicle	202	14.916±0.371	26	15	/	/	/
	27-HMA-100 μM	178	13.708±0.354	23	15	-8.10	-11.54	P > 0.05

Notes: Lifespan experiments were analyzed using Kaplan-Meier survival analysis and compared among groups, scoring for significance using the log-rank test. All data were expressed as mean± SEM. ***p < 0.001 vs. control group.

Supplementary Table S7. Effects of 27-HMA on the paralysis of CL4176 *C. elegans* induced by Aβ.

Strains	Group	Number	Mean Survival ± SEM (hours)	Median survival time (hours)	Increase in mean survival (%)	p value of mean survival
CL4176	Vehicle	136	54.221±0.783	54	/	/
	27-HMA-100 μM	136	59.500±0.832	60	9.76	P < 0.01**
CL2120	Vehicle	116	68.601±3.300	72	/	/
	27-HMA-100 μM	108	84.926±3.314	96	23.8	P < 0.01**
CL4176	Vehicle	104	60.302±0.601	60	/	/
(L4440)	27-HMA-100 μM	106	63.226±0.582	64	4.85	P < 0.01**

CL4176	Vehicle	164	62.030±0.447	64	/	/
(*nhr-8* RNAi)	27-HMA-100 μM	111	61.054±0.665	60	-1.57	P > 0.05
CL4176	Vehicle	103	59.272±0.721	57	/	/
(*daf-12* RNAi)	27-HMA-100 μM	104	59.587±0.683	57	0.53	P > 0.05

Notes: Paralysis assays were analyzed using Kaplan-Meier survival analysis and compared among groups, scoring for significance using the log-rank test. All data were expressed as mean± SEM. **p < 0.01 vs. control group.

Supplementary Table S8. List of primers for quantitative real-time PCR in *C. elegans*

Gene	Forward primer	Reverse primer
β-actin	TCGGTATGGGACAGAAGGAC	CATCCCAGTTGGTGACGATA
daf-2	TCGAGCTCTTCCTACGGTGT	CATCTTGTCCACCACGTGTC
age-1	CCTGAACCGACTGCCAATC	GTGCTTGACGAGATATGTGTATTG
daf-16	TCAAGCCAATGCCACTACC	TGGAAGAGCCGATGAAGAAG
sod-2	AAGTTCAATGGAGGAGGAC	GACAGTTGATGCCGAAAG
sod-3	TGGCTAAGGATGGTGGAG	TTGAACCGCAATAGTGAT
hsp-16.2	CTGCAGAATCTCTCCATCTGAGTC	AGATTCGAAGCAACTGCACC
sir-2.1	GCAAGAAATAACGGAGGA	TTTGAGCACGACGAAGAT
cyp-13a7	AAAAATGGCAATGGGACAAG	AATACTTTGAATATCGGTAG
cyp-13a11	GCAAATTCTCGCCGTTGTAT	TCGTCTCCTGATTCCCATCT
cyp-14a1	CCTTTCTTGGGGTCTCATCA	AAGTAGCGGCTTGGATTGAA
cyp-14a3	CAGGCACTGGAGACAAATCA	CAGGCACTGGAGACAAATCA
cyp-35a1	CGGAGTCACTGTTGCTCAAGCC	AGACTTCAAACGCAGCACCCATG
cyp-35a2	ACTGGTGGCATTGTTTCGACTCTC	GGAATTGGTCCGACCCATAGTGTG
cyp-35a3	GCTCAACTCAGTGCTCTCCATGTC	TCCCAGGCAACTTCTCTTTCCAAC
cyp-35a4	CTGACCGTGCTTCAACTCCATACC	TCCAGCATCGACAGGGTGACC
cyp-35a5	GGGAAGGAGCCGATGGAAATCAAG	GGGAAGGAGCCGATGGAAATCAAG
cyp-35b1	TGAACACGAGATGTGCCGAA	AACGTTTTCCGACGAGCAGA
cyp-35b2	GTTCCTCCCGCCTGTTTTCT	TTTCCTCGCATCTTGCATCC
cyp-35b3	GTGATTATGAAACGTCGCAAGAAG	GCGGATGCTGTAAATGGAAAGAC
cyp-35C1	AAAGTGACTAACGGAGGATCTCG	CTAGCAAGAGCCGAGCTGTATTT
cyp-36a1	GGTGGAAGGCTCAACGACGATTC	GCCAACGAAGCAATTGTGTCCTG
gst-4	CCAAATGGAGTCGTTGGCTTC	TTTGATGCTCGTGCTCTTGC
gst-10	GGAGTCCGCGATGTTCGTAT	TTCACTAGAGCCTCCGGGAT
ugt-44	GCACATTTTGGTATGCTCTGCT	CGGCAACAGAAGGGTCACAT
pgp-3	GTGATGGGACTTCCTGACGG	CTTTGGGTCTCTGACAATCGC
pgp-12	CCACTCATGTACCACGGCAT	AATAGCATTCCAGCGGCAGT
pgp-13	CCGATGGCATAGACACCGAA	GCTTCTTGCACAGCCCTTTC
pgp-14	AGGAGTACGGTGCTAGCGAT	ACATCTTTGGGGCGTCATCA
